# Supplementary material for: Computational estimates of mechanical constraints on cell migration through the extracellular matrix
Source: PLoS Comput Biol. 2020 Aug 27;16(8):e1008160. doi: 10.1371/journal.pcbi.1008160 (PMC7480866; doi:10.1371/journal.pcbi.1008160)
Supplement: S1 Text — Sections S1 and S2 describe the fluid dynamics and numerical methods used to solve the model equations. The remeshing algorithm used to simulate mechanism 2 is described in Section S3. Section S4 contains 16 figures showing the cell and ECM position at several time values for the parameter regimes listed in Table 1. An analysis of the sensitivity of the results to changes in nuclear size and bending rigidity of the cortex/nucleus is also described in Section S4. (PDF) [file pcbi.1008160.s001.pdf]

# Supporting Text

# Computational estimates of mechanical constraints on cell migration through the extracellular matrix

Ondrej Maxian, Alex Mogilner, and Wanda Strychalski

## S1. Fluid dynamics

For cellular motility problems, small length scales (microns) and slow movements (microns per minute) lead to extremely small Reynolds numbers [1], and the fluid permeating the cell and ECM obeys the Stokes equations:

$$\mu \Delta \boldsymbol{u} - \nabla p = -\boldsymbol{f} \tag{S1.1}$$

$$\nabla \cdot \boldsymbol{u} = 0. \tag{S1.2}$$

Here $\mu$ is the fluid viscosity, $p$ is the pressure, $\boldsymbol{u}$ is the fluid velocity, and $\boldsymbol{f}$ is an external force (which comes from any immersed solid structures). For simplicity, we assume that the nucleoplasm, cytoplasm and interstitial fluids all have the same viscosity.

*S1.1. Solution method: regularized Stokeslets*

Because the Stokes equations are linear, boundary integral methods can be used to solve the system in Eqs. (S1.1) and (S1.2). In particular, the solution to the Stokes equations in free space can be determined by convolving the external force function $\boldsymbol{f}$ against the free space Green's function for Stokes flow over the interface or immersed structure where the force is applied [2]. However, the singularity of the Green's function at the point where the force is applied must be treated with accurate quadrature rules, or the singular integral must be regularized. We choose to regularize the problem with the method of regularized Stokeslets [3]. In particular, we suppose that the external force of strength $\hat{\boldsymbol{F}}_0$ is distributed over a small ball centered on a point $\boldsymbol{x}_0$ (rather than confined exclusively to the point $\boldsymbol{x}_0$), so that

$$\boldsymbol{f}(\boldsymbol{x}) = \hat{\boldsymbol{F}}_0 \phi_\epsilon(\boldsymbol{x} - \boldsymbol{x}_0). \tag{S1.3}$$

The solution to the Stokes equations with external forcing given by Eq. (S1.3) can be derived from the form of the "blob" or "cutoff" function $\phi_\epsilon$. For example, if

$$\phi_\epsilon(\boldsymbol{x}) = \frac{3\epsilon^3}{2\pi(\|\boldsymbol{x}\|^2 + \epsilon^2)^{5/2}}, \tag{S1.4}$$

then

$$p^\epsilon(\boldsymbol{x}, \boldsymbol{x}_0) = \frac{1}{2\pi}(\hat{\boldsymbol{F}}_0 \cdot (\boldsymbol{x} - \boldsymbol{x}_0)) \left( \frac{r_0^2 + 2\epsilon^2 + \epsilon\sqrt{r_0^2 + \epsilon^2}}{(\sqrt{r_0^2 + \epsilon^2} + \epsilon)(r_0^2 + \epsilon^2)^{3/2}} \right), \tag{S1.5}$$

and

$$\begin{aligned} \boldsymbol{u}^\epsilon(\boldsymbol{x}, \boldsymbol{x}_0) = &-\frac{\hat{\boldsymbol{F}}_0}{4\pi\mu} \left( \ln\left( \sqrt{r_0^2 + \epsilon^2} + \epsilon \right) - \frac{\epsilon(\sqrt{r_0^2 + \epsilon^2} + 2\epsilon)}{(\sqrt{r_0^2 + \epsilon^2} + \epsilon)\sqrt{r_0^2 + \epsilon^2}} \right) \\ &+ \frac{1}{4\pi\mu}(\hat{\boldsymbol{F}}_0 \cdot (\boldsymbol{x} - \boldsymbol{x}_0))(\boldsymbol{x} - \boldsymbol{x}_0) \frac{\sqrt{r_0^2 + \epsilon^2} + 2\epsilon}{(\sqrt{r_0^2 + \epsilon^2} + \epsilon)^2 \sqrt{r_0^2 + \epsilon^2}} \end{aligned} \tag{S1.6}$$

are the pressure and velocity that result from the force in Eq. (S1.3), where $r_0 = \|\boldsymbol{x} - \boldsymbol{x}_0\|$. The derivation of these expressions can be found in [3]. An equivalent formulation is to write the result of Eq. (S1.6) as a matrix-vector product, so that

$$\boldsymbol{u}(\boldsymbol{x}) = \mathbf{M}(\boldsymbol{x}, \boldsymbol{x}_0)\hat{\boldsymbol{F}}_0, \tag{S1.7}$$

where the matrix $\mathbf{M}$ is determined from the locations of the points $\boldsymbol{x}$ and $\boldsymbol{x}_0$.

The key point here is that given the external forcing term(s) $\hat{\boldsymbol{F}}_0$, the velocity at any point $\boldsymbol{x}$ in the domain can be determined by Eq. (S1.7). The units in Eq. (S1.6) imply that the "force" $\hat{\boldsymbol{F}}_0$ has units pN/$\mu$m.

### *S1.2. Stokes' paradox and force imbalances in 2D*

A complication arises when modeling objects that have nonzero net forcing in 2D. Here we wish to consider an extracellular matrix (ECM) composed of fibers that are tethered together by springs. In that case, it is virtually impossible for the net force on the ECM to be zero during a dynamic simulation, where by definition the fibers are moving and the resulting forces are changing. A net force on the domain inevitably results from our model of the ECM. The fundamental solutions of Stokes flow in free space (regularized in Eq. (S1.6)) contain a logarithmic term so that net force imbalances result in velocities as $||\boldsymbol{x}|| \to \infty$. Thus the "usual" free space boundary condition of zero velocity at $\infty$ is incompatible with the net forcing, and a new boundary condition must be imposed [4]. One option is to specify a rigid ECM, which leads to a no-flow boundary condition on the surface of the ECM fibers. Here we study the effect of ECM elasticity and want to account for fiber flexibility. We therefore follow our previous work [5] and impose the boundary condition of *zero mean flow on a large circle of radius $R$ that surrounds the domain.* As $R \to \infty$, this boundary condition is equivalent to the usual free space boundary condition. We use the value of $R = 1000$ (cell diameters) as in [5].

The boundary condition on the large circle of radius $R$ allows for net nonzero forcing within the domain and can be enforced by adding a constant velocity throughout the domain of flow. In [5], we derived this velocity for a single point force as

$$\boldsymbol{u}^R(\boldsymbol{f}_0) = -\frac{\hat{\boldsymbol{F}}_0}{4\pi\mu}\left(\frac{1}{2} - \ln R\right). \tag{S1.8}$$

If there are $N$ points where force is applied, each with force $\hat{\boldsymbol{F}}_k$, the constant velocity added throughout the domain is

$$\boldsymbol{u}^{\boldsymbol{R}} = \sum_{k=1}^{N} \boldsymbol{u}^R\left(\hat{\boldsymbol{F}}_k\right) = -\sum_{k=1}^{N} \frac{\hat{\boldsymbol{F}}_k}{4\pi\mu}\left(\frac{1}{2} - \ln R\right). \tag{S1.9}$$

This ensures that the additional boundary condition is satisfied and yields a well-posed problem.

The full numerical scheme is therefore as follows: given a collection of $N$ points with applied forces $\hat{\boldsymbol{F}}_k$, Eq. (S1.7) is used to compute an intermediate velocity at each of the $N$ points. Then the boundary condition is enforced through the addition of a constant velocity in Eq. (S1.9), so that the total fluid velocity at each point is the sum of the two. Explicit first-order time-stepping is then used to update the positions of the points.

One of the numerical parameters in the model is $\epsilon$, the width of the blob function in hydrodynamic Eq. (S1.4). As discussed in [3] and [6], $\epsilon$ should be on the order of the spacing of the grid in the system. We set $\epsilon = 0.075$ cell diameters.

## S2. Elastic forces

In this section, we describe how forces due to stretching and bending on the cortex and nucleus are implemented in our numerical algorithm.

Defining notation first, we denote by $\boldsymbol{X}_m$ the $m$th point on a fiber $\boldsymbol{X}$; i.e. $\boldsymbol{X}_m = \boldsymbol{X}(s_m)$, where $s$ is a parameterization in reference arclength coordinates and $m = 1, \ldots N$. Any function $\phi$ is defined at the $m$th point by $\phi_m$. The reference point corresponding to $\boldsymbol{X}_m$ is defined as $\boldsymbol{X}_m^0$.

We begin by defining a central difference operator to approximate the (reference arclength) derivatives along the periodic fiber [7]

$$D_s\phi = \frac{\phi_{m+1/2} - \phi_{m-1/2}}{\left\|\boldsymbol{X}^0_{m+1/2} - \boldsymbol{X}^0_{m-1/2}\right\|}. \tag{S2.1}$$

All indices are interpreted in the periodic sense, so that if $m = N$, the number of fiber points, the index $m+1$ maps to index 1. In the case of an equispaced reference configuration, the denominator of Eq. (S2.1) becomes $\Delta s = 2\pi r/N$, where $r$ is the radius of the contour in its circular configuration.

For the case of elastic energy due to **stretching**, we define the tangent vectors as

$$\boldsymbol{\tau}_{m+1/2} = \frac{\boldsymbol{X}_{m+1} - \boldsymbol{X}_m}{\left\| \boldsymbol{X}^0_{m+1} - \boldsymbol{X}^0_m \right\|}, \tag{S2.2}$$

and the tension is

$$T_{m+1/2} = k_{m+1/2}\left(\left\|\boldsymbol{\tau}_{m+1/2}\right\| - r_{m+1/2}\right). \tag{S2.3}$$

Notice that $k_{m+1/2}$ and $r_{m+1/2}$ are the tension constant and rest length at $s_{m+1/2}$. In mechanism 2, these change as a function of the position on the fiber. The unit tangent vector $\boldsymbol{\tau}$ is dimensionless and $k$ has units of pN/$\mu$m. The elastic force density at point $m$ in pN/$\mu$m$^2$ is then defined as [7]

$$\boldsymbol{F}^{\mathrm{el}}_m = \frac{T_{m+1/2}\boldsymbol{\tau}_{m+1/2} - T_{m-1/2}\boldsymbol{\tau}_{m-1/2}}{\left\| \boldsymbol{X}^0_{m+1/2} - \boldsymbol{X}^0_{m-1/2} \right\|}. \tag{S2.4}$$

To obtain a force density in pN/$\mu$m from force density in pN/$\mu$m$^2$, we multiply by $\left\| \boldsymbol{X}^0_{m+1/2} - \boldsymbol{X}^0_{m-1/2} \right\|$,

$$\widehat{\boldsymbol{F}}^{\mathrm{el}}_m = T_{m+1/2}\boldsymbol{\tau}_{m+1/2} - T_{m-1/2}\boldsymbol{\tau}_{m-1/2}. \tag{S2.5}$$

The calculation for equispaced reference nodes is the same, but the denominator of $\boldsymbol{\tau}_{m+1/2}$ can be simplified to $\Delta s$ in that case.

We model **bending** energy with a preferred curvature. Let the cortex/nucleus radius in the reference state be given by $r$. We model the preferred curvature $\kappa = 1/r$. The bending energy (units of pN$\cdot\mu$m) is then defined as [7],

$$E_b = \frac{1}{2}\sum_{m=1}^{N} K_{b,m}\left(\left\|D_s D_s \boldsymbol{X}_m\right\| - \kappa\right)^2 \left\| \boldsymbol{X}^0_{m+1/2} - \boldsymbol{X}^0_{m-1/2} \right\|. \tag{S2.6}$$

In 2D simulations, we define the bending constant to have dimensions pN$\cdot\mu$m. The notation $K_{b,m}$ indicates the bending constant depends on the node index. For mechanism 2, the bending constant can be zero in regions where the cortex is loose and relaxed, and its value can be nonzero where the cortex is stiff.

Carrying out the numerical differentiation in Eq. (S2.1) twice, we have

$$D_s D_s \boldsymbol{X}_m = \frac{1}{\left\| \boldsymbol{X}^0_{m+1/2} - \boldsymbol{X}^0_{m-1/2} \right\|}\left(\frac{\boldsymbol{X}_{m+1} - \boldsymbol{X}_m}{\left\| \boldsymbol{X}^0_{m+1} - \boldsymbol{X}^0_m \right\|} + \frac{\boldsymbol{X}_{m-1} - \boldsymbol{X}_m}{\left\| \boldsymbol{X}^0_{m-1} - \boldsymbol{X}^0_m \right\|}\right). \tag{S2.7}$$

Differentiating the (negative of the) energy with respect to $\boldsymbol{X}_\ell$, we have the force density (in pN/$\mu$m),

$$\hat{\boldsymbol{F}}^b_\ell = \sum_{m=1}^{N} \frac{K_{b,m}}{\left\| \boldsymbol{X}^0_{m+1/2} - \boldsymbol{X}^0_{m-1/2} \right\|^2}\left(\frac{\boldsymbol{X}_{m+1} - \boldsymbol{X}_m}{\left\| \boldsymbol{X}^0_{m+1} - \boldsymbol{X}^0_m \right\|} + \frac{\boldsymbol{X}_{m-1} - \boldsymbol{X}_m}{\left\| \boldsymbol{X}^0_{m-1} - \boldsymbol{X}^0_m \right\|}\right)\left(1 - \frac{\kappa}{\left\|D_s D_s \boldsymbol{X}_m\right\|}\right) \tag{S2.8}$$

$$\left(-\frac{\delta_{m+1,\ell}}{\left\| \boldsymbol{X}^0_{m+1} - \boldsymbol{X}^0_m \right\|} - \frac{\delta_{m-1,\ell}}{\left\| \boldsymbol{X}^0_{m-1} - \boldsymbol{X}^0_m \right\|} + \frac{\delta_{m,\ell}}{\left\| \boldsymbol{X}^0_{m+1} - \boldsymbol{X}^0_m \right\|} + \frac{\delta_{m,\ell}}{\left\| \boldsymbol{X}^0_{m-1} - \boldsymbol{X}^0_m \right\|}\right)\left\| \boldsymbol{X}^0_{m+1/2} - \boldsymbol{X}^0_{m-1/2} \right\|.$$

Unlike for elastic forces, the denominator $\left\| \boldsymbol{X}^0_{m+1/2} - \boldsymbol{X}^0_{m-1/2} \right\|$, which is unknown, no longer vanishes. We therefore approximate

$$\boldsymbol{X}^0_{m+1/2} - \boldsymbol{X}^0_{m-1/2} \approx \frac{1}{2}\left(\boldsymbol{X}^0_{m+1} - \boldsymbol{X}^0_{m-1}\right), \tag{S2.9}$$

so that the force in pN/$\mu$m at node $\ell$ is given by

$$\widehat{\boldsymbol{F}}_\ell^b = \sum_{m=1}^{N} K_{b,m} \frac{2D_sD_s\boldsymbol{X}_m}{\|\boldsymbol{X}^0_{m+1}-\boldsymbol{X}^0_{m-1}\|}\left(1-\frac{\kappa}{\|D_sD_s\boldsymbol{X}_m\|}\right) \tag{S2.10}$$
$$\left(-\frac{\delta_{m+1,\ell}}{\|\boldsymbol{X}^0_{m+1}-\boldsymbol{X}^0_{m}\|}-\frac{\delta_{m-1,\ell}}{\|\boldsymbol{X}^0_{m-1}-\boldsymbol{X}^0_{m}\|}+\frac{\delta_{m,\ell}}{\|\boldsymbol{X}^0_{m+1}-\boldsymbol{X}^0_{m}\|}+\frac{\delta_{m,\ell}}{\|\boldsymbol{X}^0_{m-1}-\boldsymbol{X}^0_{m}\|}\right).$$

For the case of equispaced nodes in the reference configuration, Eq. (S2.10) simplifies to

$$\widehat{\boldsymbol{F}}_\ell^b = \sum_{m=1}^{N} \frac{K_{b,m}}{\Delta s^4}\left(\boldsymbol{X}_{m-1}-2\boldsymbol{X}_m+\boldsymbol{X}_{m+1}\right)\left(1-\frac{\kappa\Delta s^2}{\|\boldsymbol{X}_{m-1}-2\boldsymbol{X}_m+\boldsymbol{X}_{m+1}\|}\right)\left(-\delta_{m+1,\ell}-\delta_{m-1,\ell}+2\delta_{m,\ell}\right), \tag{S2.11}$$

which is the form of [7, Eq. (17)], modified to include a preferred curvature.

## S3. Remeshing algorithm

Here we test our remeshing algorithm outside of the cell motility simulations. We consider a closed elastic fiber (representing the cell cortex or nuclear envelope) of radius 1 discretized with $N = 50$ points, sitting by itself in a domain of fluid of viscosity $\mu = 1$ Pa·s inside and outside. The back half of the cell (points $\lceil N/4 \rceil$ to $\lfloor 3N/4 \rfloor$) is stiff with $k = 100$ pN/$\mu$m and resting spring length $r = 0.5$, while the front of the cell (blue circles) is loose, with $k = 1$ pN/$\mu$m and $r = 1$ (these constants refer to the tension equation, (4) in the main text). These parameters are similar as those used in the mechanism 2 simulations, with the exception that we choose here to keep $r = 0.5$ so that the dynamics of the back of the cell are similar to those shown in mechanism 2 simulations. Elastic forces using a remeshed configuration are computed as described in Section S2. The key ingredient is to resample the reference configuration of the nodes, so that deviations from the original circular configuration (with all nodes equispaced) are penalized by the elastic forcing.

We simulate the dynamics of the circle within the method of regularized Stokeslets with $\epsilon$ equal to the initial point spacing. We begin by simulating the cell to steady state ($t = 7$ with $\Delta t = 0.01$) without remeshing. Fig. S1(a) shows the deformed shape at this point. The original back half of the cell (points $\lceil N/4 \rceil$ to $\lfloor 3N/4 \rfloor$ marked with red diamonds) has collapsed down, leaving the front-half potentially unresolved. Fig. S1(b) shows the corresponding arclength function $s(j)$ at this point in the algorithm as a blue line. From indices 13 to 37 ($\lceil N/4 \rceil$ to $\lfloor 3N/4 \rfloor$), the slope of the arclength function changes drastically because the points are more tightly packed. In Fig. S1(b), the new *sample locations* are shown as red circles. These locations are more sparse in the region of decreased slope. This indicates that the arclength function $s(j)$ is being sampled at equally spaced node indices, $S\left(j^{\text{eq}}\right)$. Indeed, as shown in Fig. S1(c), the points $\boldsymbol{X}(S\left(j^{\text{eq}}\right))$ are equally spaced on the cell after they are remeshed (we always keep the 2 points bound to the ECM nodes). Finally, Fig. S1(d) shows the new reference configurations, $\boldsymbol{X}^0(S\left(j^{\text{eq}}\right))$, which become more sparse at the back of the cell.

The remesher always includes the two points bound to ECM nodes. Because of this, an ideal test for the remesher is to consider the displacement of one of these points with and without remeshing. That is, we vary the number $N$ of points on the cortex and simulate to $t = 7$ (empirically determined to be steady state) with $\Delta t = 0.01$ as before. Next, we simulate to $t = 8$ with and without remeshing (only once at $t = 7$) and determine how much the first red point in the second quadrant (moving counterclockwise) has moved relative to not remeshing (i.e., the error is the norm of the position of the point at $t = 8$ with remeshing minus the position without remeshing). Table S1 shows the results of this test. It appears that our remesher makes an error on the order $\mathcal{O}(10^{-4})$ for $\approx 100$ points, which is what we use in our simulations. This error could be decreased by increasing the number of points.

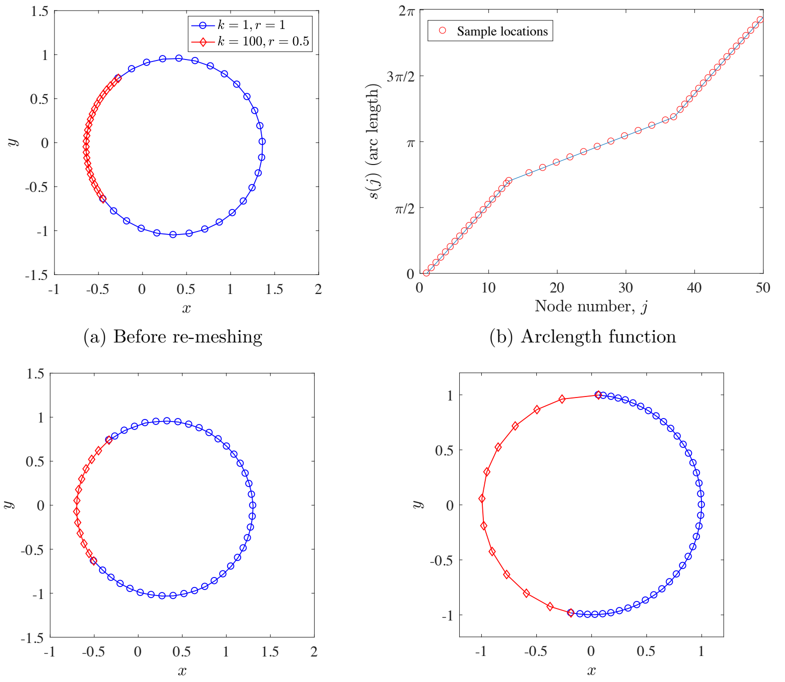


(a) Before re-meshing

(b) Arclength function

(c) After re-meshing

(d) New reference locations

Figure S1: Test on the remeshing algorithm with $N = 50$ points. (a) Cell before remeshing. The back of the cell (red diamonds) is rigid with $k = 100$ pN/$\mu$m and $r = 0.5$, while the front of the cell (blue circles) is looser with $k = 1$ pN/$\mu$m and $r = 1$. (b) The arclength function $s(j)$ is sampled at equal distances to give the new node locations, which are interpolated from the old locations. (c) After remesh. We note how the points are now evenly distributed. The point of interest in the error calculation is the first counter-clockwise red point in the second quadrant of the cell. (d) The new reference locations used to calculate the elastic force after remeshing.

The procedure for remeshing the nucleus is exactly the same as that described for the cortex, with the exception that there are no “bound points” that must be preserved by the remesher. Thus the sampling of the arclength function is truly uniform in this case.

| $N$ | 25 | 50 | 100 | 200 |
|---|---|---|---|---|
| Error | $5.5 \times 10^{-3}$ | $1.6 \times 10^{-3}$ | $4.1 \times 10^{-4}$ | $1.2 \times 10^{-4}$ |

Table S1: Errors in the final position of the first red node in the second quadrant (in counterclockwise order).

## Supporting References


[1] A. Mogilner, A. Manhart, Intracellular fluid mechanics: coupling cytoplasmic flow with active cytoskeletal gel, Annu. Rev. Fluid Mech. 50 (2018) 347–370. doi:0.1146/annurev-fluid-010816-060238.

[2] C. Pozrikidis, Boundary integral and singularity methods for linearized viscous flow, Cambridge University Press, 1992.

[3] R. Cortez, The method of regularized Stokeslets, SIAM J. Sci. Comput. 23 (4) (2001) 1204–1225. doi:10.1137/S106482750038146X.

[4] G. Morra, Insights on the Physics of Stokes Flow, in: Pythonic Geodynamics. Lecture Notes in Earth System Sciences, Springer, 2018, pp. 93–104.

[5] O. Maxian, W. Strychalski, 2D force constraints in the method of regularized Stokeslets, Comm. App. Math. Com. Sc. 14 (2) (2019) 149–174. doi:10.2140/camcos.2019.14.149.

[6] R. Cortez, L. Fauci, A. Medovikov, The method of regularized Stokeslets in three dimensions: analysis, validation, and application to helical swimming, Phys. Fluids 17 (3) (2005) 031504. doi:10.1063/1.1830486.

[7] L. Zhu, C. S. Peskin, Simulation of a flapping flexible filament in a flowing soap film by the immersed boundary method, J. Comput. Phys. 179 (2) (2002) 452–468. doi:10.1006/jcph.2002.7066.

## S4. Supporting Figures

The figures in this section show the position of the cortex (green), nucleus (magenta), and ECM nodes (blue) at several time values during a simulation. Time values increase from left to right and top to bottom for figures in this section. Key parameter values for each simulation are indicated in the figure captions using bold text. See Table 1 in the main text for exact parameter values.

### *S4.1. Push-pull mechanism of motility*

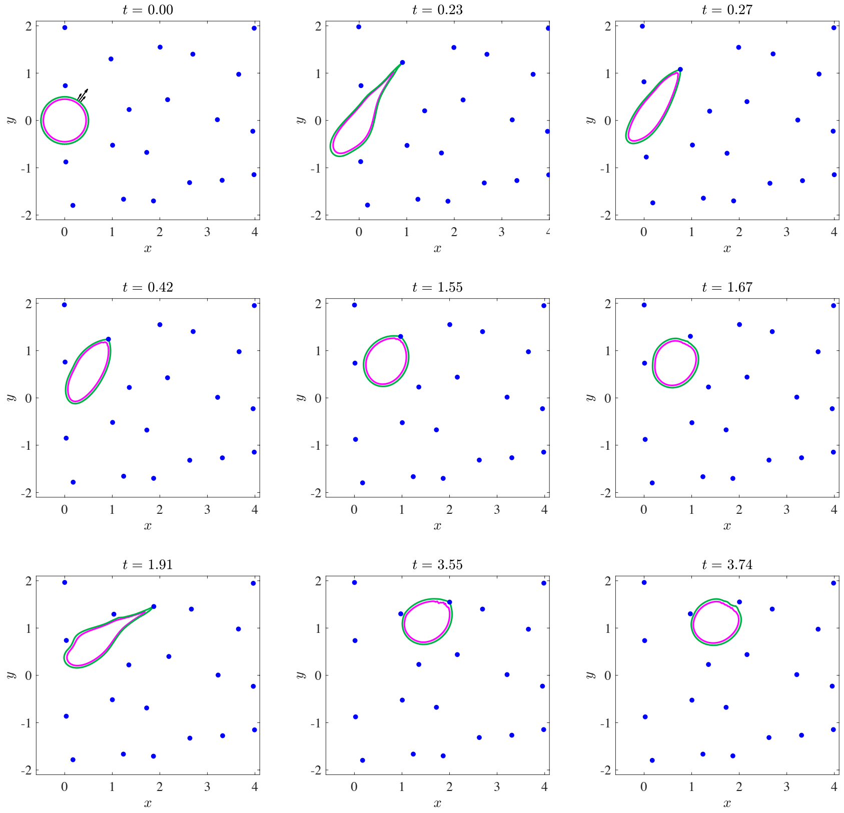


Figure S2: **Sparse ECM**: The cell migrates easily through the sparse network (same as the network shown in Fig. 1(a) of the main text).

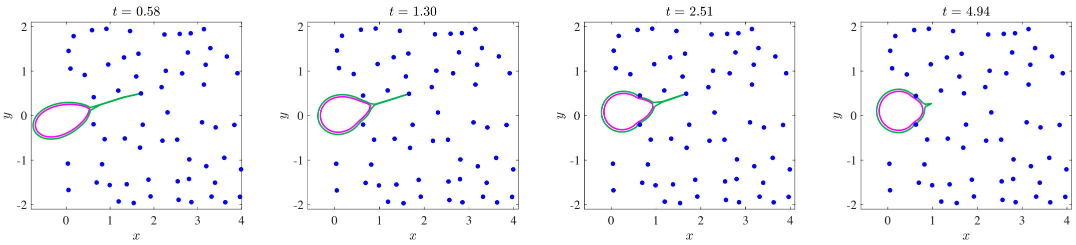

Figure S3: $\boldsymbol{E} > \boldsymbol{T}$ **and** $\boldsymbol{N} > \boldsymbol{T}$. The cell becomes stuck in the ECM during the simulation.

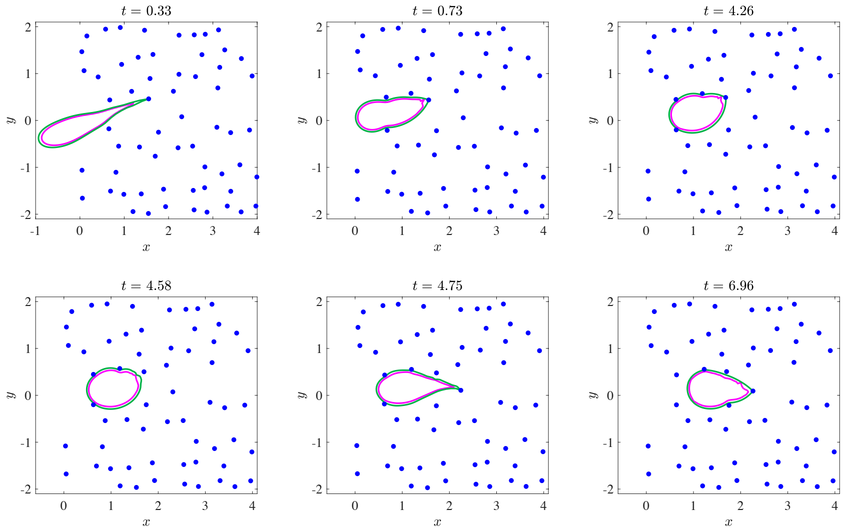

Figure S4: $\boldsymbol{T} > \boldsymbol{E} > \boldsymbol{N}$: The cell migrates by squeezing the nucleus, which is relatively more deformable than the cortex and ECM.

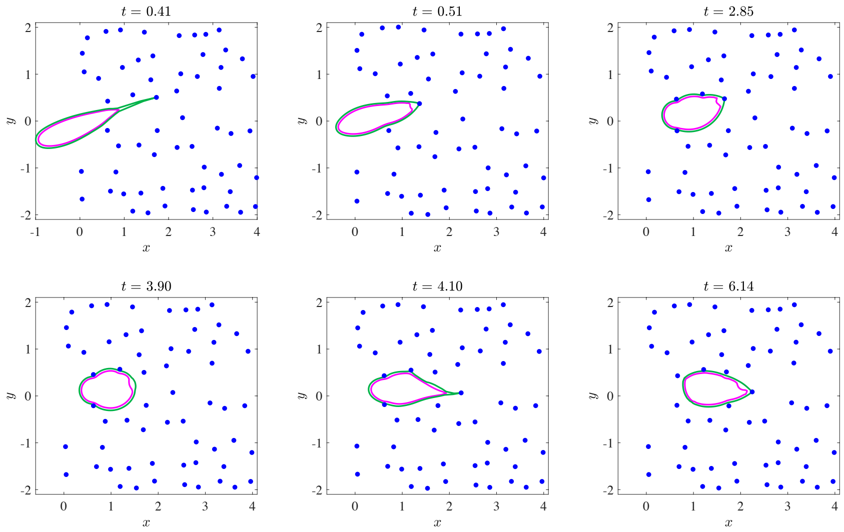

Figure S5: $\boldsymbol{T} > \boldsymbol{N} > \boldsymbol{E}$. The cell moves using a combination of nuclear and ECM deformation.

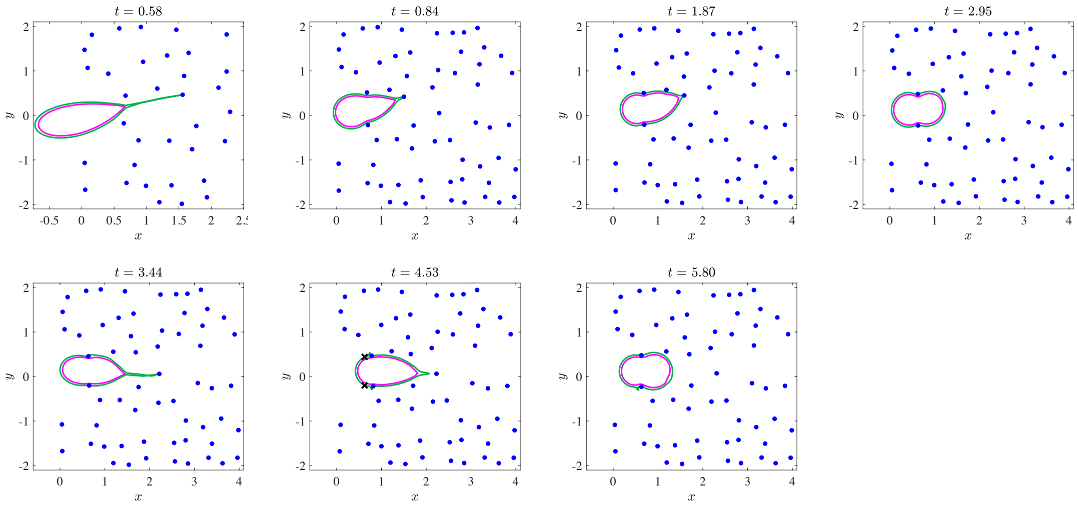


Figure S6: $\boldsymbol{N} > \boldsymbol{T} > \boldsymbol{E}$: The cell moves by a combination of nuclear and ECM deformation. However, in this parameter regime, the cell must deform the ECM to migrate because the nucleus is relatively stiffer (compare to the data in Fig. S7). Black $\times$'s are used to show the initial position of the key ECM nodes, which achieve their maximum displacement at $t = 4.53$.

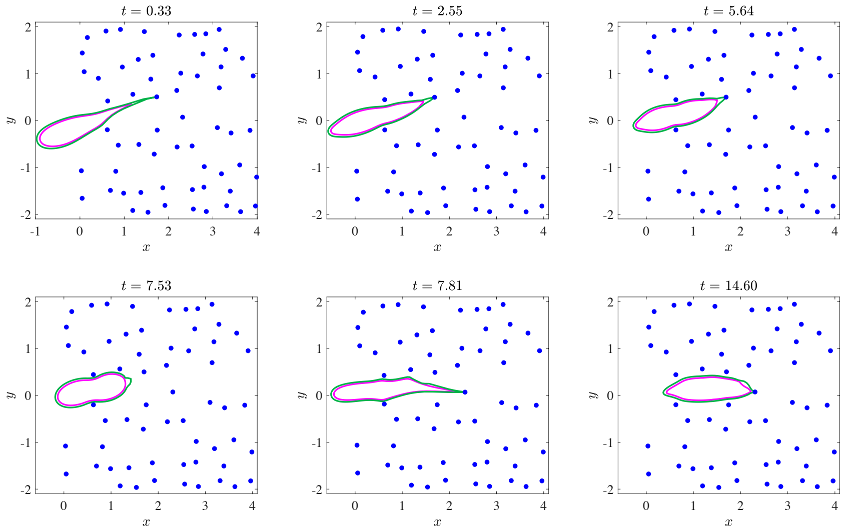


Figure S7: $\boldsymbol{E} > \boldsymbol{T} > \boldsymbol{N}$: The cell moves by squeezing the nucleus because of relatively reduced tension in the cortex. Note the elongated cell shape for this parameter regime.

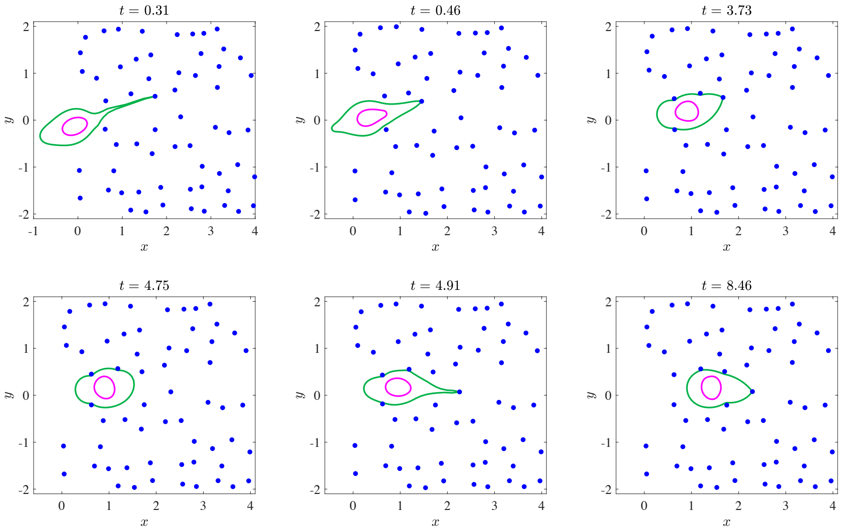

Figure S8: ***N* > *T* > *E* for a small nucleus**: We use the same parameters as in Fig. S6, but halve the diameter of the nucleus. The behavior is qualitatively more similar to the $T > N > E$ regime shown in Fig. S5. Thus halving the nuclear size simply decreases the characteristic force $N$ required to deform the nucleus.

*Quantifying migration distance*

We quantify the distance moved by the nucleus and the amount that the nucleus and cortex penetrate the ECM. The nuclear displacement is measured by tracking the mean of the coordinates on the nuclear contour (the nucleus is centered at the origin at the start of the simulation). In Figs. S4–S9, the cell begins migrating by moving through a gap between two ECM nodes. We quantify penetration into the network by measuring the fraction of nucleus and cortex points that pass through this gap (past the two nodes). Table S2 shows these statistics at the end of the first motility cycle.

The data in Table S2 show that halving the diameter size of the nucleus with the $N > T > E$ parameters, i.e., keeping all other parameters fixed at $\tilde{k}^{c,r} = 50$, $\tilde{k}^{n} = 100$, results in a larger distance moved and more penetration into the network. While the nuclear penetration is larger (since the nucleus is smaller), the total distance moved by the nucleus is similar to the $T > N > E$ regime with the original larger nucleus. We conclude that halving the nuclear diameter for mechanism 1 is equivalent to reducing the force required to move the nucleus.

| Parameters | Distance | Nucleus penetration | Cortex penetration |
|---|---|---|---|
| $T > E > N$ | 1.12 | 0.88 | 0.80 |
| $T > N > E$ | 0.97 | 0.78 | 0.70 |
| $N > T > E$ | 0.66 | 0.51 | 0.49 |
| $^*N > T > E$ | 0.92 | 1.00 | 0.69 |
| $E > T > N$ | 0.58 | 0.45 | 0.43 |

Table S2: Distance traveled after the first cycle in Figs. S4–S8 computed by tracking the nucleus' center of mass. Penetration is calculated using the fraction of points on the nucleus and cortex that move past the line dividing the two ECM nodes that the cell moves past, approximately located at the points $(0.5, \pm 0.5)$. Data indicated by $^*$ are simulated with a small nucleus ($r_{\text{nuc}} = 0.225$).

*Bending rigidity*

We determine the effect of bending rigidity by performing simulations using the same parameters as in Table S2, but including a finite bending rigidity of $K_b = 0.05$ (and preferred curvature of a circle) for both the nucleus and cortex. For the cortex, we introduce the bending energy only after the cell binds to an ECM node.

Table S3 shows the percentage change for distance and penetration when bending energy is included. We see that the measurements are generally within ±10% of the data from Table S2, with the exception of $E > T > N$. Surprisingly, the distance increases when bending rigidity is included for this regime. Simulation results in Fig. S9 show the cortex becomes rounder at the front and back. Increased migration distance results when the round cortex at the leading edge fills out the empty space and pulls the nucleus forward. Rounding at the cell rear also pushes the nucleus through the gap between the ECM nodes.

| Parameters | Distance | Nucleus Penetration | Cortex penetration |
|---|---|---|---|
| $T > E > N$ | −4% | −4% | −5% |
| $T > N > E$ | +1% | 0% | +2% |
| $N > T > E$ | +12% | +10% | +8% |
| $E > T > N$ | +37% | +25% | +21% |

Table S3: Distance and penetration data for mechanism 1 with bending energy included on both the nucleus and cortex ($K_b = 0.05$, preferred curvature of a circle). Bending energy on the cortex is only included after the cell binds to an ECM node, while it is always included on the nucleus. Percentages are the percentage change from the data in Table S2.

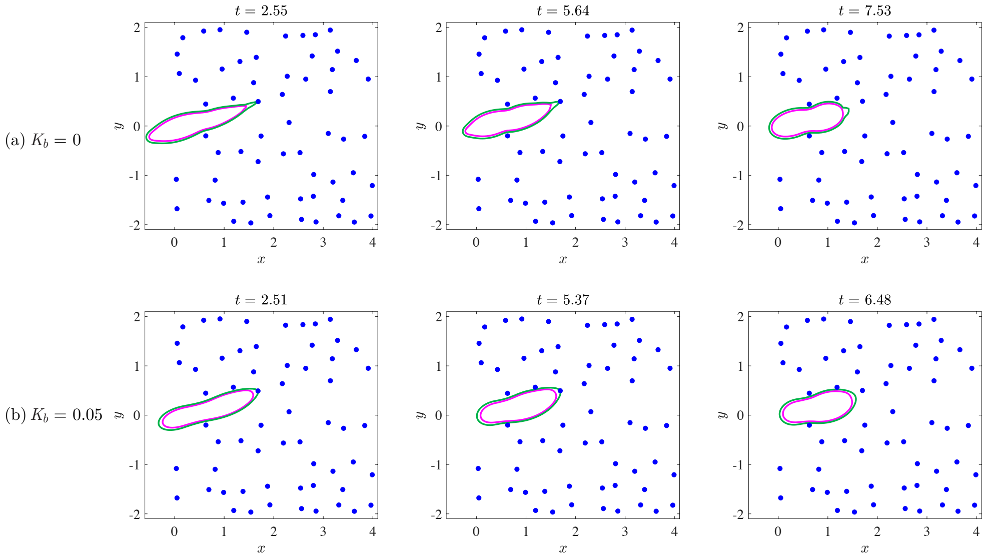


Figure S9: **Varying the bending energy using mechanism 1 in the parameter regime $E > T > N$.** (a) Simulation results with no bending energy (where the parameter $K_b = 0$), and (b) results at comparable time values to (a) where the bending stiffness parameter was increased to $K_b = 0.05$ throughout the entire nucleus, and in the cortex after binding to an ECM node. Distance traveled increases when bending is included for this parameter regime. The rounder nucleus in (b) results in a decrease in $N$ relative to $T$. The rounder cortex at the leading edge increases $T$, pulling the cell forward.

### *S4.2. Rear-squeezing mechanism of motility*

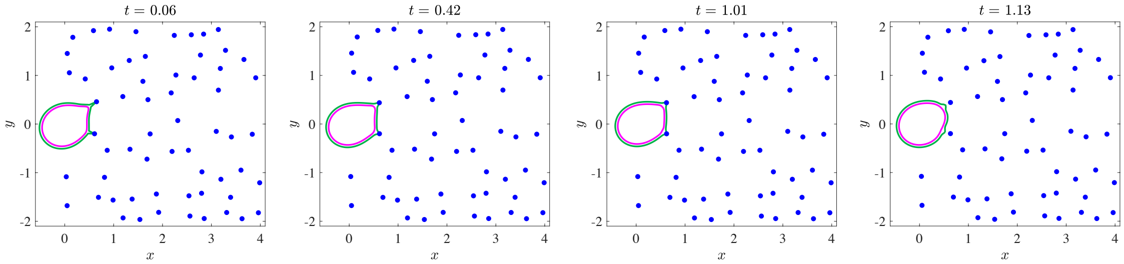


Figure S10: $\boldsymbol{E > T}$ **and** $\boldsymbol{N > T}$: When the contraction of the cell cortex is too weak to deform either the nucleus or ECM, the cell is not able to squeeze through a gap in the ECM.

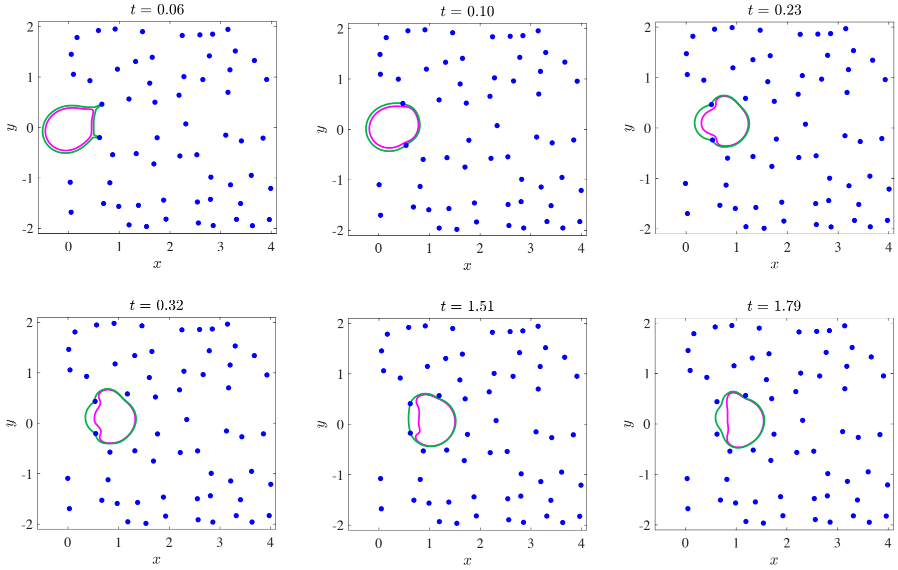


Figure S11: $\boldsymbol{T > E > N}$: The cell can easily pass through the gap between the fibers because the nucleus is soft compared to the cortex and ECM.

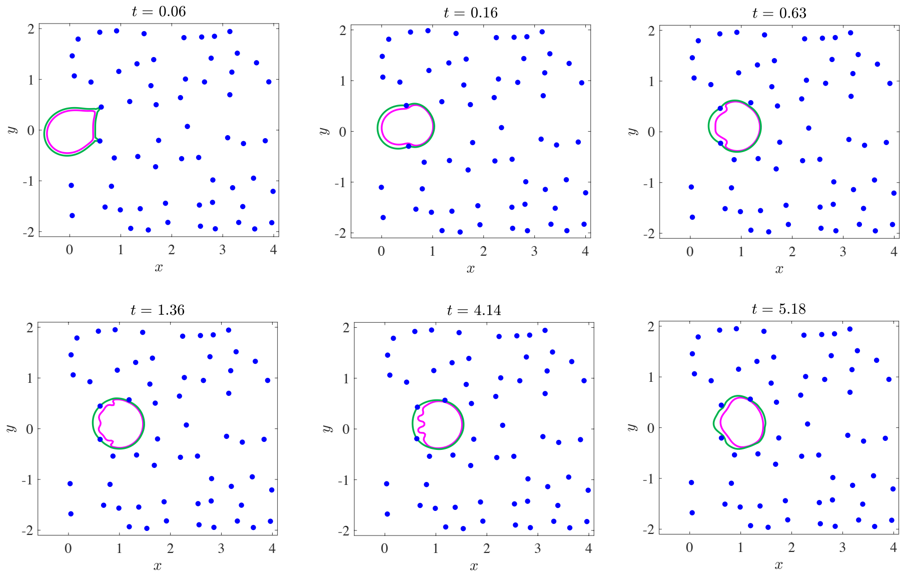


Figure S12: $\boldsymbol{T} > \boldsymbol{N} > \boldsymbol{E}$: The nucleus begins to buckle (bottom row) because of high cortical tension and increased cortical stiffness. After releasing the nodes, the nucleus partially regains its rounded shape (compare the position of the nucleus at $t = 5.18$ to $t = 4.14$).

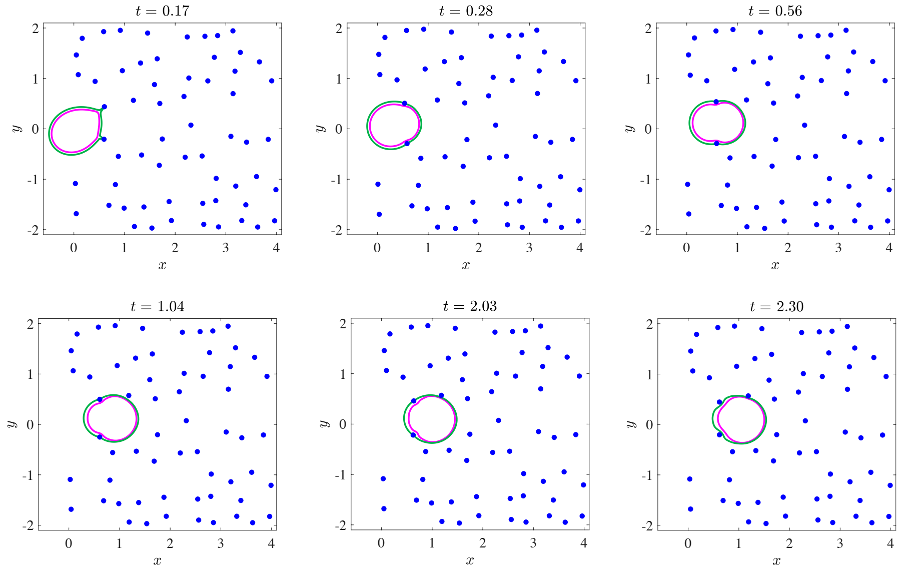


Figure S13: $\boldsymbol{N} > \boldsymbol{T} > \boldsymbol{E}$: In spite of the nucleus' relatively large stiffness, the cell is still able to migrate through the ECM. However, the cell is unable to entirely pass through the gap after one motility cycle.

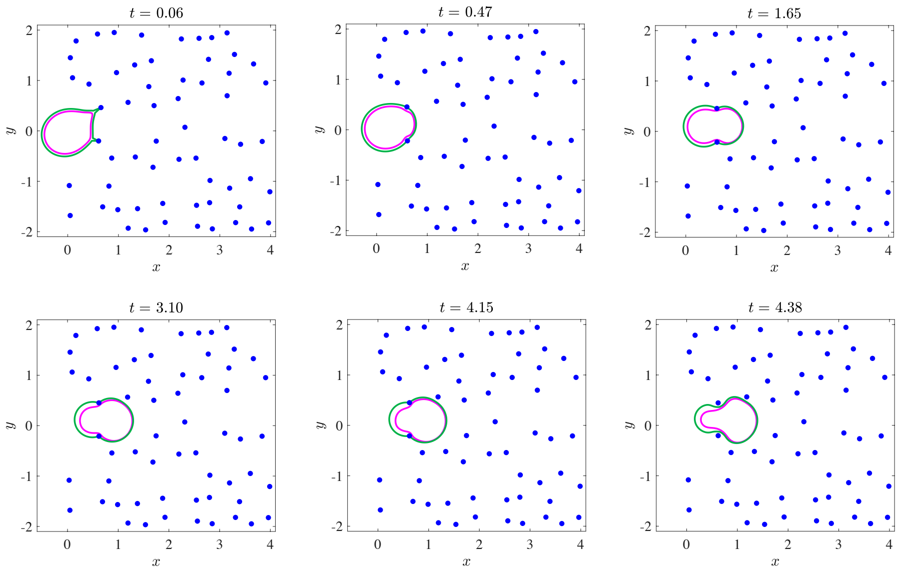

Figure S14: $\boldsymbol{E > T > N}$: Although the cell makes some progress, there is not enough force to drive it completely through the gap. After one motility cycle, the cell retracts slightly after releasing the nodes.

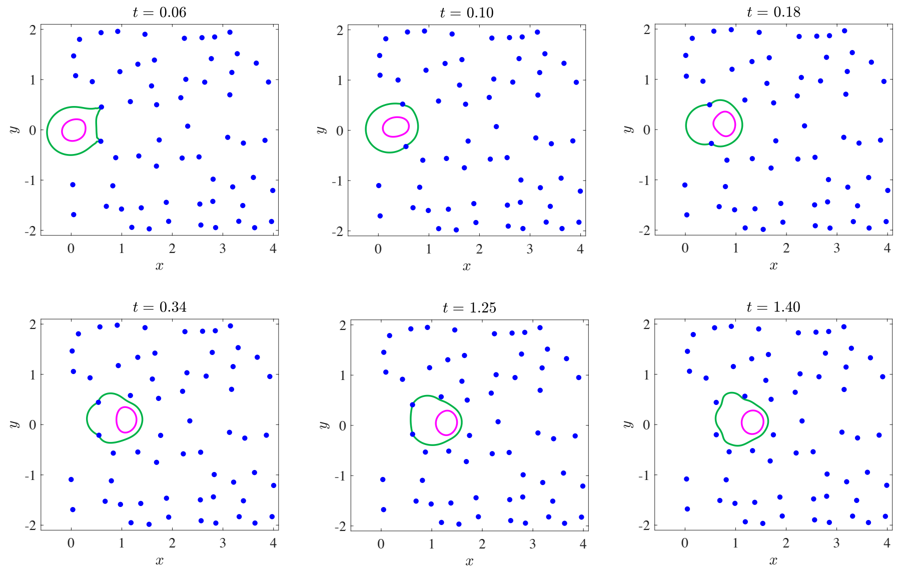

Figure S15: **$\boldsymbol{N > T > E}$ for a small nucleus**: We use the same parameters as in Fig. S13, but halve the diameter of the nucleus. The behavior is qualitatively more similar to the $T > E > N$ regime shown in Fig. S11.

*Quantifying migration distance*

As in the previous section, we quantify the distance traveled and the penetration of the nucleus and cortex into the ECM network. We measure distance (nuclear displacement) by computing the center of mass of the nuclear contour (since the nucleus is centered at the origin at the start of the simulation). In Figs. S11−S16, the cell begins migrating by binding to two ECM nodes and moving through the gap between them. We quantify penetration into the network by measuring the fraction of nucleus and cortex points that pass through this gap (past the nodes). Table S4 shows these data at the end of the first motility cycle.

The nucleus travels a larger distance when its size is reduced. Data in Table S4 show that halving the nuclear diameter while keeping the parameters from the $N > T > E$ regime in mechanism 2 ($\tilde{k}^{c,r} = 100$, $\tilde{k}^{n} = 1000$) results in behavior more characteristic of the $T > E > N$ regime in simulations with the original larger nucleus. In mechanism 2, decreasing the diameter allows the nucleus to move more easily because the fluid from the back of the cell is able to push the nucleus forward.

| Parameters | Distance | Nucleus penetration | Cortex penetration |
|---|---|---|---|
| $T > E > N$ | 1.12 | 1.00 | 0.92 |
| $T > N > E$ | 1.11 | 0.94 | 0.83 |
| $N > T > E$ | 1.06 | 0.90 | 0.78 |
| $^{*}N > T > E$ | 1.34 | 1.00 | 0.94 |
| $E > T > N$ | 0.88 | 0.71 | 0.65 |

Table S4: Distance traveled after the first cycle in Figs. S11−S15 computed by tracking the nucleus' center of mass. Penetration is calculated using the fraction of points on the nucleus and cortex that move past the line dividing the two ECM nodes approximately located at the points $(0.5, \pm 0.5)$. Data indicated by $^{*}$ are simulated with a small nucleus ($r_{\text{nuc}} = 0.225$).

*Bending rigidity*

We analyze the importance of bending rigidity for mechanism 2 by including a finite bending rigidity of $K_b = 0.05$ on both the nucleus and cortex (with preferred curvature of a circle). Bending energy is included in the cortex in the stiff cell rear after it binds to the two nodes. Other simulation parameters are the same as used to generate data in Table S4. Table S5 shows the relative distances travelled with bending stiffness are approximately the same as those in Table S4, with the exception of the regime $E > T > N$. We note this is the same parameter regime where bending forces cause increased distance traveled in mechanism 1. In contrast to mechanism 1, motility decreases when the nucleus has bending energy in mechanism 2. Data in Fig. S16 show the nucleus is unable to deform enough to squeeze through the gap between the ECM nodes. The qualitative behavior of the cell with bending rigidity is similar to simulations without bending rigidity in the parameter regime $E > N > T$ where migration fails.

| Parameters | Distance | Nucleus penetration | Cortex penetration |
|---|---|---|---|
| $T > E > N$ | −3% | −10% | −11% |
| $T > N > E$ | −7% | −13% | −8% |
| $N > T > E$ | +3% | +11% | +4% |
| $E > T > N$ | −61% | −56% | −50% |

Table S5: Distance and penetration data for mechanism 2 with bending energy included on both the nucleus and cortex ($K_b = 0.05$, preferred curvature of a circle). Bending energy on the stiff part of cortex is only included after the cell binds to an ECM node, while it is always included on the nucleus. Percentages are the percentage change from the data in Table S4.

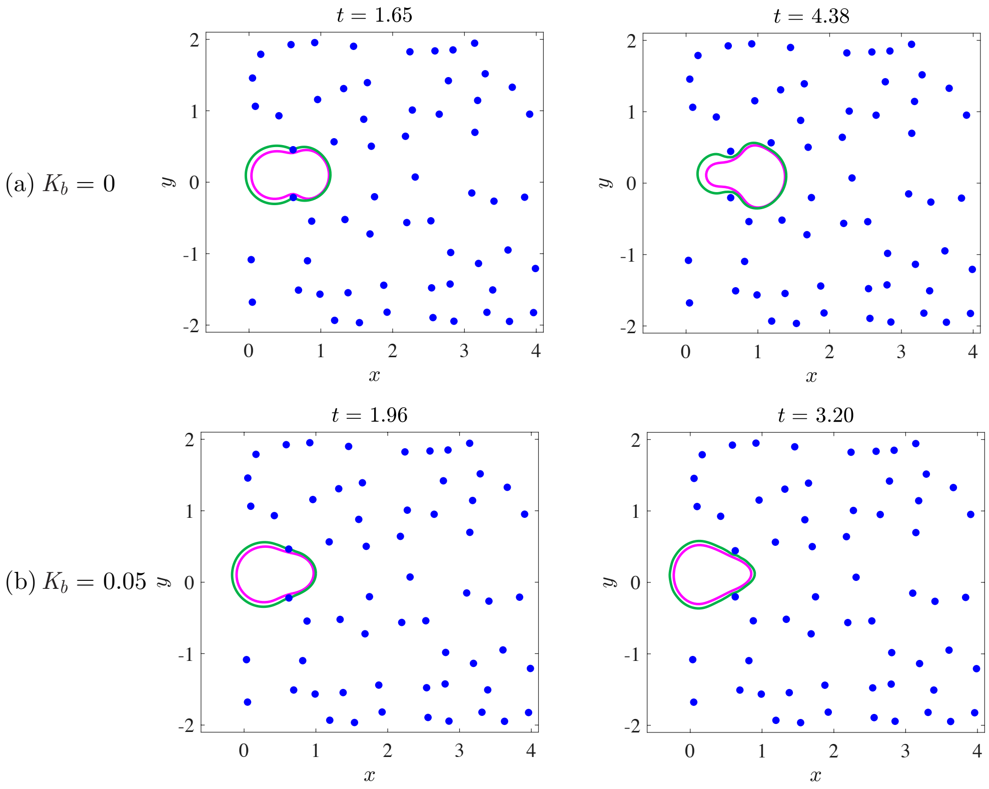


Figure S16: **Varying the bending energy using mechanism 2 in the parameter regime $E > T > N$.** (a) Simulation results with no bending energy (where the parameter $K_b = 0$), and (b) results at comparable time values to (a) where the bending stiffness parameter was increased to $K_b = 0.05$ in the stiff (rear) region of the cortex and throughout the entire nucleus. Increasing the strength of the force due to bending increases the nuclear force (represented by the parameter $N$) and prevents the cell from moving through the ECM. Results are similar to those from the regime $E > N > T$, where the cell was unable to migrate through the ECM.
