# Supplementary material for: Computational estimates of mechanical constraints on cell migration through the extracellular matrix
Source: PLoS Comput Biol. 2020 Aug 27;16(8):e1008160. doi: 10.1371/journal.pcbi.1008160 (PMC7480866; doi:10.1371/journal.pcbi.1008160)
Supplement: Attachment — Submitted filename: ReviewerResponses.pdf [file pcbi.1008160.s029.pdf]

Computational estimates of mechanical constraints on cell migration through the extracellular matrix

Ondrej Maxian, Alex Mogilner, and Wanda Strychalski

We thank the reviewers for their thoughtful and constructive comments. We have substantially revised the manuscript accordingly. Edits in revised paper in response to specific comments are color coded: blue text for reviewer 1, red text for reviewer 2, and green text for reviewer 3. Some of our methods have changed in response to reviewers comments, and new data was obtained that slightly changed some of our results. Text that has been removed or added as a result of the new data is colored in gray. References, citations, and page numbers in the following responses match the revised paper.

Reviewer #1

The article propose a computational model to describe cell migration in a 3D fibrous environment, which is different from the motion on a two-dimensional substratum, focusing in particular on the effect of several mechanical aspects, such as the contractile force acting on the cortex, the elasticity of the nucleus and the rigidity of the ECM. The main aim is to describe the difference between mesenchymal and ameboid modes of migration. This is a subject that is recently attracting a lot of attention.

1.1 In the abstract, the Authors' Summary and the introduction it is stressed that the model is three-dimensional, so I was expecting a 3D cell. Actually, the computational environment is a slice of a three-dimensional set-up, as correctly stated at the beginning of the section on the qualitative model description. Justifying this simplification with axisymmetry arguments or indefinite extension in the third dimension is unrealistic. I think that the authors should simply state that their computational domain is the mid-plane of the cell and, above all, anticipate the statement to the abstract and/or to the introduction.

On p. 4, in the first paragraph of "qualitative model description," we removed the attempts to justify the 2D simplification and simply state that we "approximate the dynamics as occurring in the mid-plane of the cell." We also added short statements in the abstract and at the end of the introduction on p. 3 to clarify that the model is two-dimensional.

1.2 Working in the framework of sub-cellular element models, the model consists in discretizing the cell and the nuclear membrane in a series of elements connected by spring with stiffness that can dynamically change according to the mode of migration. In this respect the work is similar to those by Bates' group (some cited) who however focus on bleb formation and don't devote much attention to the mechanical aspects of migration. On the other hand, the work by Scianna et al. (Math. Biosci. Engng. 10, 235–261 (2013)) use a cellular Potts model focusing on the effect of the stiffness of the nucleus and of the extra-cellular matrix on cell migration mainly in a 3D environment with a regular and random distribution of ECM fibers. For this reason, I think that these results should be mentioned. I also find some similarities with the approach used by Schmeiser's group (see, for instance, J. Math. Biol. 74 (2017), pp. 169-193). So a comparison would be desirable.

We thank the reviewer for drawing our attention to these papers. We added respective references to the Introduction with comments about insights from these studies.

Modelling

1.3 Generally speaking, I think that more details on the dynamics of the membrane should be given in the main text, so that the differences between the two migration modes can be more clearly related to the modelling terms and parameters.

We rewrote the modeling description on pp. 7-10 for clarity and added additional information about the migration modes. In particular, we added a step by step description of each mechanism so readers can easily compare them.

ECM

1.3 The ECM is treated as a set of points connected by springs that in Eq. (1) seem to have vanishing rest length. An artificial addition of an extra-force (pinning-down force) is needed to ensure that the configuration is in equilibrium. Then, it is stated that this force is mimicking the effect of the resting length of the springs. Why not writing more correctly the mechanics of the springs with finite rest lengths given by the distance set between points? Then one could linearize the force to get Eq. (1). On the other hand, this would easily allow to introduce, if wanted, the highly nonlinear behaviour of ECM to strong deformations.

Although including spring rest lengths in the model may be more physically relevant, including resting lengths without the pinning-down force can result in translations of the entire lattice. Since we are modeling the cell migration in relation to an ECM network, additional forces are necessary to maintain the overall network structure so that the whole network does not significantly deform. The force arising from including a spring resting length could be included in a straight-forward manner by redefining $\hat{\boldsymbol{F}}^{ECM,0}(\boldsymbol{X}_i)$ *in Eq. (1). We modified the text on p. 5 below on Eq. (1) to clarify the purpose of the 'pinning down' force.*

1.5 From the simulations it seems that the ECM actually reacts like a viscoelastic material (a sort of creep behaviour) rather than an elastic one. Where does this viscous behaviour comes from? From the cell fluid?

Indeed, we observe effective Kelvin-type viscoelastic behavior of the ECM, where the effective viscous and elastic elements are in parallel. When a constant stress is applied to the network, its deformation exponentially approaches the deformation of a pure elastic material, with the viscosity of the fluid determining the relaxation timescale. In our model, the viscosity arises from the fluid surrounding the ECM as well as inside the cell and nucleus. We clarify this point in the paragraph below **Extracellular matrix** *on p. 5 of the revised paper.*

Mesenchymal migration

1.6 Regarding mesenchymal migration, some aspects of the dynamics should be described better, e.g., how is the protrusion generated? (randomly? Cells are not polarized and there is no chemotaxis, right? Or there is a predefined front and back?)

In mesenchymal migration, the cell randomly generates a protrusion in the front half of the cell, i.e. facing the positive x *direction. Thus, in our model, the cell has already polarized with a predefined front and back. We do not specifically include chemotaxis in our model, but it is plausible that a chemical signal could induce cell polarization prior to migrating. We modified the text on the bottom of p. 7 to clarify that the cell generates random protrusions with a preferred migration direction.*

1.7 Is it possible to generate more protrusions as it actually occurs in cells? Why is this not done? In the movie successive spiking

Unfortunately it appears some of the comment here is missing. Our goal here is to consider the mechanics of the cell's motion in the ECM, assuming that it forms a protrusion which binds to the nodes. One could assume that this is the first protrusion that binds out of several simultaneous protrusions. That said, we did consider the case of unsuccessful protrusions that retract without attaching to ECM nodes; this is discussed at the end of the mechanism 1 and 2 sections on pp. 9-10, with an additional comment on the bottom of p. 9 to clarify that unsuccessful protrusions are frequent in real cells. This point is also addressed in the second paragraph of the conclusion.

1.8 Frankly speaking, the dynamics generated looks a bit strange because of the contemporary backward extension. Is this related to the fluid dynamics inside the cell?

Indeed, the backward extension is related to the fluid dynamics. The cell cannot exert a net force on the fluid. The "forward" pointing force on the protrusion therefore has to be balanced by the backward pointing force on the rest of the cell. Thus the front of the cell moves forward while the back slowly lurches backward (prior to the protrusion binding). We added a comment on p. 7 after Eq. (6) to clarify this point.

Ameboid migration

1.9 Regarding ameboid migration, the fact that the two protrusions that are generated with an angle smaller than 45 degrees find anchoring points nearby depends on the location of ECM points. What happens if adhesion points are not found?

If adhesion points are not found, the cortex contour stiffens globally and the protrusions retract. This is addressed in the last paragraph of the "Mechanism 2: Rear-squeezing mode" section on p. 10.

1.10 Is the stiffening homogeneous in the front and in the rear of the cell? Is it graded? Time-dependent? How is all this set? How is the splitting between region identified? What is distinguishing in the model the front pulling and the rear pushing? The fact that there is no f_0?

The distinguishing factor between the two mechanisms is the stiffening region. In mechanism 1, one protrusion binds to a node and the cortex stiffens globally until the cell's velocity drops below a threshold value. This is discussed in the third paragraph of "Mechanism 1: push-pull mesenchymal mode", beginning on p. 7, and continuing on p. 9. For mechanism 2, only part of the cortex contour stiffens. In particular, the region behind the nodes stiffens while the front remains loose, which pushes the nucleus from behind. This is addressed in the second paragraph of "Mechanism 2: Rear-squeezing mode" section that begins on p. 9 and continues to p. 10. The stiff and loose regions are also discussed for each mechanism in the new list of steps provided on pp. 9-10.

Minor points

1.11 Why the x-axis range between 0 and 4 and the y-axis range between -2 and 2.

We wanted to show symmetry in the y direction and movement in the x direction. Since we are concerned about the absolute distance traveled and the direction of motion is $+x$*, we chose to initially center the cell at the point* $(0, 0)$ *and move rightwards.*

1.12 Though an experimental article is cited, I'm really afraid that the volume of the cytoplasm is really small. There are pictures from Friedl's group related to 3D experiments where this volume is larger and in my opinion more realistic. Due to the compartimentalization between cytoplasm and nucleus, this might have strong impact on the fluid-dynamics inside the cytoplasm.

We added simulations with a small nucleus to the Supporting Text in Section S4 for both mechanisms of motility. We use the same parameters as the $N > T > E$ *regime for both mechanisms and halve the diameter of the nucleus. Simulation results are shown in Figs. S8 (mechanism 1) and S15 (mechanism 2). We also added a discussion to explain that our simulations show that reducing the nuclear size simply changes the magnitude of the nuclear force* N *(located on p. 10 (mechanism 1) and p. 15 (mechanism 2) of the Supporting Text).*

One of the reasons we chose such a large nucleus is to be able to model the regime $N > T > E$*, where the force due to nuclear tension is relatively large. Indeed, if the nucleus is small, the nuclear force* N *does not limit the cell's ability to migrate through the ECM network.*

To clarify that our focus is on the strengths between the forces and not the size of the nucleus, we added comments at the beginning of the mechanism 1 (p. 14) and 2 (p. 17) results sections in the main text.

Conclusion

I think that there are some points to be clarified and some more details on the modelling to be given, which requires major revisions before the article can be published.

We thoroughly revised the paper to address the reviewer's concerns, including extensive clarification of our methods and new simulations with a small nucleus.

Reviewer #2

The authors have developed a 2D computational framework to simulate cell migration through a fibrous environment by two different mechanisms: mesenchymal (push-pull) and amoeboid (rear-squeezing). The cell is divided into two compartments (nuclear and cytoplasmic), each is represented by a closed contour, and fluid flow is simulated in both of these compartments and in the extracellular environment using the boundary integral method (regularized Stokeslets in 2D, a standard approach for flow around microswimmers or obstacles). Because of the hydrodynamic equations, the contours cannot cross. Forces by nucleus (N), cell (T) and matrix (E) are represented by spring constants. The main results are that for successful migration, either $T > N$ or $T > E$ is required, but not both. More importantly, large N is a more severe limitation than large E. The authors also show that deformation energy stored in N or E as well as pressure in the fluid can aid migration.
The general conclusions drawn from this study are very interesting and certainly of interest to the large field working on cell migration. The paper is written and presented very well. The strong focus on nuclear mechanics is very timely.

We thank the reviewer for carefully reading our paper and articulating the important points of our study.

However, while the motivation and conclusions are clear and interesting, the actual model is not very transparent and has many issues. In the following, I list the main issues that I see.

We thoroughly revised the paper by including substantial clarification of our methods. We include simulations with a small nucleus in the revised paper as well as simulations to study the effect of forces due to bending for both the nucleus and cortex.

2.1 Nucleus: nuclear size is fixed very close to cell size. The simulated cells do not look like real cells, the nucleus is very large and often appears to act more like a cortex. In fact it is modeled not as a bulk material, but as a hollow shell. There is also concern that the fluid flow between the two close-by contours is not represented well if they are so close, because then the cutoff function of the regularized Stokeslets might become relevant. In my view, the authors have to present also simulations with a smaller nucleus, because only then can the reader judge how well the method (in particular the hydrodynamics) works.

We thank the reviewer for pointing out this issue. We have performed simulations with a smaller nucleus in the Supporting Text, Section S4. Please refer to the last response to reviewer #1 for more information on the smaller nucleus. In short, we added a discussion on Supporting Text p. 10 and p. 15 about how our results change with a smaller nucleus.

They also should show some examples of fluid flow during deformations (not only the pressure field).

We modified Fig. 5 to show the pressure, velocity, and speed of the fluid during migration. We included both a failing and successful parameter regime for both mechanisms to show how these quantities change with improved motility.

2.2 Volume conservation: it is not clear to me if and how volume (area in the 2D model) is kept constant.

The area is kept approximately constant during an entire simulation since the fluid flow field is divergence-free. There is some numerical error from simulating the model equations, but it is on average only about 5%. We added a comment in the model description on p. 4 to clarify this point.

2.3 Flow boundary conditions: it is not explained which boundary conditions result from the Stokeslet method (if any). Traditionally one would implement no-slip boundary conditions, at least in the normal direction. Can fluid mix between compartments? Is pressure continuous across the boundaries as suggested by Fig. 5? What are the absolute values predicted by this theory and how does this compare to experimental results, e.g. in the context of blebbing?

We included further clarification on these points in the revised paper on p. 4. In particular, we clarify that the fluid cannot mix between compartments due to fluid incompressibility and impermeability of

the cortex and nucleus. Indeed, a no-slip boundary condition is imposed at the fluid-contour interfaces. The regularized Stokeslet method assumes free space boundary conditions for the fluid velocity at infinity.

The pressure field is technically continuous on the length scale of the contour regularization (represented by the parameter ϵ, see Section S1 in the Supporting Text). On larger length scales, such as the diameter of the cell, pressure jumps can occur. For example, when there is an active tension across the cell membrane, pressure is discontinuous across the membrane. This is the case for the rear of the membrane in mechanism 2, and we added a comment on p. 21 (in the discussion of Fig. 5) on this point. The pressure jump in the cell rear shown in Fig. 5 II (b) is approximately 100 Pa, the same order of magnitude as experimentally measured in a blebbing cell from [35].

2.4 Viscosities: if we disregard the interaction of water with ECM-components, we expect an interstitial viscosity close to the one of water. Cytoplasmic viscosity on the scale of the cell is known to be many orders of magnitude higher than the one of water due to the presence of the cytoskeleton. Nuclear viscosity is expected to be even higher than the one of the cytoplasm due to high chromatin density. Thus I wonder why the three viscosities are all set to the same value here. Can the Stokeslet method be extended to different viscosity values and what would be the main consequences?

We chose all the fluid viscosities to be the same value for simplicity. While it is possible to simulate variable viscosities with the method of regularized Stokeslets, it requires the introduction of the regularized Stresslet and is much more involved to simulate. For example, having viscosity ratios as large as 10 can introduce additional numerical stiffness and increases the simulation time considerably. We added text on p. 12 to clarify this point.

We acknowledge that including different viscosities for different compartments would be an interesting topic for future studies. We expect including different viscosities for the cytoplasm, nucleoplasm, and extracellular fluid will primarily affect the overall migration time scale rather than the cell shape and migration strategy.

2.5 Elasticity: cell and nuclear stiffness are represented by springs in the contour and there is neither a bulk elasticity nor a bending energy of the contour. This leads to many issues. The simulated shapes often have cusps that would not appear in real cells. I suggest to make also simulations with bending energy. Also there should be more discussion why bulk elasticity is not modeled and how realistic the conversion of bulk values to spring constants really is.

For a fully 3D model, the cell membrane can be modeled by a 2D neo-Hookean elastic shell that has a bulk (stretching) modulus and bending modulus. In a 2D model with a 1D contour representing the cell, the force due to the bulk or stretching modulus becomes the stretching force that we refer to as $\boldsymbol{F}^{el}$*. We tried to revise the text to clarify that the force* $\boldsymbol{F}^{el}$ *is not simply a bunch of springs tying the points together, but a discretization of an elastic continuum energy.*

To investigate the effects of including bending rigidity, we performed simulations with forces arising from a bending energy as described in the new Section S2 of the revised Supporting Text. We added a discussion of these simulations in the Supporting Text, Section S4, p. 11 (mechanism 1) and p. 15 (mechanism 2).

2.6 Matrix: to make the model more predictive, the authors should simulate different matrix densities and compare to literature results on how cells cope with intermediate versus high fiber densities (I agree that sparse is not interesting in this context). Maybe I have overlooked this, but it appears that density is not really varied, although this might be the most rewording strategy to compare with experiments. Like in Figs. 3 and 4, it would be interesting to see cell speed as a function of density.

As the reviewer points out, we varied the ECM density between "sparse" (when it is easy for the cell to move around) and "dense" (when the cell is blocked from moving). Having a dense ECM allows us to analyze the fundamental parameter regimes that characterize the cell motion. The ECM force E *has magnitude equal to the ECM spring constant times the distance the ECM has to deform when the cell moves through it. In the present study, we chose to fix the magnitude of* E *and varied* N *and* T *around this value to analyze the six different orderings of* E, N, *and* T*. We feel that varying* E *is*

somewhat counter-productive, since our simulations were designed with a fixed value of E. We note that introducing an intermediate density ECM has the same effect as decreasing E relative to N and T.

2.7 Comparison between the two migration modes: the authors find that the rear-squeezing mode is more efficient, but this might be mainly due to their specific rule that two rather than one protrusion is used here. What happened if the other mechanism is implemented with two protrusions ? How can the cell control protrusions at the front by a mechanism that acts at the back ? Obviously this mode requires more regulation than the push-pull mode. There is concern here that the rear-squeezing mode is better by construction.

As we already mentioned in the Discussion, we only investigate the mechanical constraints on the cell migration, not the kinetic ones. We expanded on this point in the Discussion as follows: Specifically, our conclusion that the rear-squeezing motility performs better than the push-pull mechanism is limited to the mechanical performance of the cell, not taking into account the time needed to extend protrusions in the right directions and establish respective adhesions with the ECM fibers. For the rear-squeezing motility, more than one such successful protrusion/adhesion processes have to occur, compared to just one in the push-pull mechanism, and it is entirely possible that these processes take much longer time in the rear-squeezing motility. In addition, differences between efficiencies of signaling processes regulating the protrusion/adhesion activity at the cell rear versus front are unknown.

2.8 Elastic forces: equation 3 is not clear to me. What is tau – the tangent vector? The derivative of the tangent vector is the normal, so this force is always normal?

We thank the reviewer for pointing out some inconsistencies in our notation. We have revised the text around Eq. (3) to try to make more clear the definition of the elastic forces. Since tension is a scalar, the tensile force density can be written as $T_s\boldsymbol{\tau} + T\boldsymbol{\tau}_s$, so that there is both a normal and tangential component. This is discussed on p. 6 of the revised text.

We also added Section S2 in the supplementary information that describes how we compute the discrete elastic and bending forces, with special attention to the case when the fiber is remeshed (in mechanism 2).

2.9 There are a few word duplications in the text ("for a for a", "the the")

We removed duplicate words in the revised paper.

Reviewer #3

The topic of this work is very interesting and important in many biological systems including cancer cell migration. I think this is a great approach to analyze cell migration mechanism in the presence of an ECM network and, basically it will deserve a publication in PLoS Comp Biol.

We thank the reviewer for recognizing the importance our of study and for assessing our work.

I have some technical questions and suggestions as follows before my strong support on this work:

3.1 Fig 2(I). Could authors add one more subpanel between figs at $t = 0.34$ and $t = 0.55$? This will provide better presentation.

We added more panels to Fig. 2(I) on p. 8 to try to show the mechanism in more detail.

3.2 Fig 2(II). I am really curious the state of cell deformation between $t = 0.17$ and $t = 0.33$. Also, It would be great to see effect of distance between two target node points on cell migration by varying this distance while fixing other ECM nodes and observing impact on cell deformation and movement patterns.

We added more panels to Fig. 2(II) on p. 8 to try to show the mechanism in more detail.

Regarding the separation between ECM nodes, we varied the ECM density between "sparse" (when it is easy for the cell to move around regardless of the nucleus/cortex parameters) and "dense" (when the cell can be blocked from moving depending on parameters). Having a dense ECM allows us to analyze the fundamental parameter regimes that characterize the cell motion. The ECM force E has magnitude equal to the ECM spring constant times the distance the ECM has to deform when the cell moves through it. In this manuscript, we chose to fix the magnitude of E and varied N and T around this value, so that we could analyze the six different orderings of E, N, and T. We feel that varying the distance between nodes (and therefore E) is somewhat counter-productive in this sense, since our design was to fix E. Decreasing the distance between nodes would increase E and hinder the movement for high N and low T, while increasing the distance will have the reverse effect.

3.3 In general, there is always an issue of transport of data with minimizing errors possible, whenever re-meshing is necessary. In this work, authors presented the error test in terms of displacement with and without remeshing (Supporting file 1). When a new mesh is created, old velocity fields and other necessary information at the nodes (or in the neighborhood of given nodes) in an old mesh must be correctly transported in a new mesh with a minimum error. For example, in continuum mechanics, whenever a new mesh is created, one would need an effective methods to minimize the errors in transporting data from the old mesh to the new mesh. I wonder how this issue was handled in this work.

We believe the reviewer is referencing adaptive FEM meshes, where information on the fine mesh has to be compatible with the same information on the coarse mesh. When we remesh the fiber, we simply obtain new Stokeslet points at which we need to compute fluid velocity. The procedure in this case is unmodified.

The challenge in our approach is in the computation of forces. Since the force is computed in reference arclength coordinates, it is important that the denominator in finite differences be handled correctly (i.e. by determining the Δs between the two reference nodes). We discuss in more detail our calculation of the forces when remeshing in the new Supplementary Section S2.

3.4 Figs. 3,4: These provide interesting interpretation of cell migration through an ECM network in various combination of E, T, N in the cases of the push-pull and rear squeezing mechanisms. Some of the results are quite consistent with experimental data. Authors provided distance information: distance/protrusion, total distance in both cases. It would be great to analyze the behavior in more detail by including subfigures of the following quantities (per each case) (i) cell volume (well area in this 2D case), (ii) ratio of the cell body lengths in two major axis (i,e., (length of the major axis in a moving direction)/(the length of the minor axis in the perpendicular direction.) (iii) portion of the cell area within the ECM network at final time in order to identify strength or magnitude of cell penetration through the ECM network.

- *(i) Because of fluid incompressibility, we expect the area inside the cortex/nucleus to stay approximately constant, as we discuss on p. 4 of the revised text. There will be numerical error, however, and so we computed the area change inside each contour. We found that the average change in the cell area for all parameter regimes is 3% for mechanism 1 and 6% for mechanism 2.*
- *(ii) We computed the aspect ratio during the simulations using Eq. (7) in the revised text. The results are shown in Fig. 3 II(c) for mechanism 1 and Fig. 4 II(c) for mechanism 2. Our conclusion is that cells with higher aspect ratios are less effective in migration.*
- *(iii) In Figs. S3-S16, the cell begins migrating by moving through a gap between two ECM nodes. We quantified penetration into the network by measuring the fraction of cortex and nucleus points that pass through this gap (past the nodes). These number are now reported in Tables S2 and S4 in the Supporting Text.*

3.5 In Fig. 5, can authors present spatial distribution of pressures and velocity fields for five cases in Table 1? If those are almost identical or very similar, it would be pointless to present this. But, I assume this will be different. If they are different, can authors explain why? This might give us more detailed information on why one mechanism is more effective than others.

When the cell successfully moves, the pressure/velocity data look similar to data in Fig. 5. We added graphs of velocity and speed to Fig. 5 in the case of successful motion (regime $T > E > N$ and unsuccessful motion (regimes $E > T, N > T$) for both mechanisms. The discussion of the revised Fig. 5 is located on p. 21.

3.6 In this work, the ECM fibers don't seem to move a lot, providing bit solid characteristics. This of course will give a migratory cell several advantages in terms of migration and penetration. What will happen if ECM density is low and the ECM network fluctuate a lot in response to given force from a cell? Would this framework still work? Or What sort of modification would be necessary in order to simulate cell migration in such a fragile ECM network?

We addressed these important questions in the Discussion. According to very few studies, which we cite, in a low-density ECM cells adopt a spindle-like morphology and migrate effectively in 1D, along an ECM fiber. On floppy ECMs, cells migrate by developing flimsy adhesions and applying very low traction forces, presumably in order not to deform the ECM too much. What, if any, effect the cell nucleus has in these cases is an open question.
