# Supplementary material for: Computational estimates of mechanical constraints on cell migration through the extracellular matrix
Source: PLoS Comput Biol. 2020 Aug 27;16(8):e1008160. doi: 10.1371/journal.pcbi.1008160 (PMC7480866; doi:10.1371/journal.pcbi.1008160)
Supplement: Attachment — Submitted filename: ReviewerResponse_v2.pdf [file pcbi.1008160.s030.pdf]

Computational estimates of mechanical constraints on cell migration through the extracellular matrix

Ondrej Maxian, Alex Mogilner, and Wanda Strychalski

We thank the reviewer for carefully reading the revised manuscript and for suggesting further clarification and improvement. Edits in the revised text are colored in blue. References, citations, and page numbers in the following responses match the revised paper.

Reviewer #2

The authors have performed major revisions on their manuscript. In my view, they have responded very well to most technical questions and the revised work is in very good shape regarding the continuum mechanics part. From the biological point of view, I appreciate the demonstration that relative stiffness of nucleus and matrix is very important for cell migration, and that hydrodynamic pressure might play a large role, favoring one mode of cell migration over another. However, I also think that the response is not sufficient yet in regard to the biological system of interest. In particular, I think that there are still two major issues that have to be addressed.

1. First they now write in the introduction “One represents the nucleus which is sometimes almost as great in size as the whole cell [31]” and later “The cell diameter is of the order 10 um, while the nuclear diameter is slightly smaller [31].” I checked Ref. 31, the PNAS paper 2009 from the Paluch group, they use fibroblasts and the nucleus here is expected to be much smaller than the cell. Actually there seem to be no data on this in that paper, but compare the cartoon in the supplement and the general literature on fibroblasts. I think that the authors should go back to the literature and give evidence for such large nuclear sizes (in my understanding, this is mainly true for stem cells, but it would also be interesting to check for white blood cells). Then they should clearly state that most model cell lines have larger cytoplasms and include new Figs. S8 and S15 (which look much more realistic than the current main figures with the large nuclei) into the main text with discussion, because this important subject should not be relegated to the supplement.

We apologize for the wrong citation. Indeed, ref [31] has no data on the nuclear size. We based our estimate on the data in:

Patricia M Davidson, Celine Denais, Maya C Bakshi, Jan Lammerding. Deformability Constitutes a Rate-Limiting Step During Cell Migration in 3-D Environments. Cell Mol Bioeng. 2014 Sep 1;7(3):293-306.

and in

Katarina Wolf, Mariska Te Lindert, Marina Krause, Stephanie Alexander, Joost Te Riet, Amanda L Willis, Robert M Hoffman, Carl G Figdor, Stephen J Weiss, Peter Friedl. Physical Limits of Cell Migration: Control by ECM Space and Nuclear Deformation and Tuning by Proteolysis and Traction Force. J Cell Biol. 2013 Jun 24;201(7):1069-84.

Davidson et al used NIH 3T3 cell line (fibroblasts); they write: "The cell nucleus ... occupies a large fraction of the cellular volume; with a typical diameter of 3–10 μm, the nucleus is larger than many of the pores encountered in the extracellular environment". In their Fig. 2, one can clearly see that the nucleus occupies a large part of the cell volume. Wolf et al. report ellipsoid nuclear shapes, 8–11 μm in diameter; they used HT/MT1 cell line (fibrosarcoma cells – metastasized fibroblasts). The characteristic volume of the fibroblast is on the order of 10^3 cubic micron, so their diameter, when they are in 3D and not too elongated is ~ 10 micron.

All this said, we appreciate the reviewer's insight; we edited the statements on pp. 3 and 10 of the revised manuscript, basically saying that we focus on interesting case when nucleus is almost as large as the whole cell, but also simulate the case when the nuclear volume is significantly smaller than that of the cell. We also followed the reviewer's advice and included data where the nuclear volume is significantly smaller than that of the cell from the Supporting Figures 8 and 15 into revised Figures 3 and 4. Additional discussion of results from the new data was added to the Results section on pp. 16 and 19.

2. Second the authors now write "The nucleus and cortex have an elastic energy, which represents a bulk modulus in three-dimensions [33]." I am not sure if I agree on this. Their elastic energy seems to be a stretching energy or cortical tension (a spring constant or line tension in 2D, surface stretching energy or surface tension in 3D), but not a real bulk energy like in a fully elastic solid. That is fine with me as a modelling choice, but should be clarified. Of course a stretching energy can be related to a 3D modulus in a thin film approximation, but this is not the point. I think the cortical buckling also comes from the fact that the authors effectively use thin shell elasticity and hydrodynamics (if the 2D model was transferred to 3D), but not bulk elasticity. It is a clear modelling choice to represent the cell by two elastic contours. Maybe I misunderstand something here, but the text is not clear yet on these important issues.

We agree and have clarified the model description on p. 4 as follows: "The nucleus and cortex have an elastic stretching energy, which is a conventional modeling choice [29, 33]; how well this approximation represents the cell mechanics is an open question. For example, other models of the cell have included a bulk elasticity throughout the cytoplasm [26, 34], which would be an extension of the model presented here."
