# Supplementary material for: Computational estimates of mechanical constraints on cell migration through the extracellular matrix
Source: PLoS Comput Biol. 2020 Aug 27;16(8):e1008160. doi: 10.1371/journal.pcbi.1008160 (PMC7480866; doi:10.1371/journal.pcbi.1008160)
Supplement: Attachment — Submitted filename: review-PLoSCompBiol-Maxian2020.docx [file pcbi.1008160.s028.docx]

The topic of this work is very interesting and important in many biological systems including cancer cell migration. I think this is a great approach to analyze cell migration mechanism in the presence of an ECM network and, basically it will deserve a publication in PLoS Comp Biol. I have some technical questions and suggestions as follows before my strong support on this work:

Fig 1(I). Could authors add one more subpanel between figs at t=0.34 and t=0.55? This will provide better presentation.

Fig 1(II). I am really curious the state of cell deformation between t=0.17 and t=0.33. Also, It would be great to see effect of distance between to target node points on cell migration by varying this distance while fixing other ECM nodes and observing impact on cell deformation and movement patterns.

In general, there is always an issue of transport of data with minimizing errors possible, whenever re-meshing is necessary. In this work, authors presented the error test in terms of displacement with and without remeshing (Supporting file 1). When a new mesh is created, old velocity fields and other necessary information at the nodes (or in the neighborhood of given nodes) in an old mesh must be correctly transported in a new mesh with a minimum error. For example, in continuum mechanics, whenever a new mesh is created, one would need an effective methods to minimize the errors in transporting data from the old mesh to the new mesh. I wonder how this issue was handled in this work.

Figs 3,4: These provide interesting interpretation of cell migration through an ECM network in various combination of E,T,N in the cases of the push-pull and rear squeezing mechanisms. Some of the results are quite consistent with experimental data. Authors provided distance information: distance/protrusion, total distance in both cases. It would be great to analyze the behavior in more detail by including subfigures of the following quantities (per each case)

(i) cell volume (well area in this 2D case),
(ii) ratio of the cell body lengths in two major axis (i,e., (length of the major axis in a moving direction)/( the length of the minor axis in the perpendicular direction.)
(iii) portion of the cell area within the ECM network at final time in order to identify strength or magnitude of cell penetration through the ECM network.

In Fig 5, can authors present spatial distribution of pressures and velocity fields for five cases in Table 1? If those are almost identical or very similar, it would be pointless to present this. But, I assume this will be different. If they are different, can authors explain why? This might give us more detailed information on why one mechanism is more effective than others.

In this work, the ECM fibers don’t seem to move a lot, providing bit solid characteristics. This of course will give a migratory cell several advantages in terms of migration and penetration. What will happen if ECM density is low and the ECM network fluctuate a lot in response to given force from a cell? Would this framework still work? Or What sort of modification would be necessary in order to simulate cell migration in such a fragile ECM network?
